# Dose-finding evaluation of once-daily treatment with olodaterol, a novel long-acting β_2_-agonist, in patients with asthma: results of a parallel-group study and a crossover study

**DOI:** 10.1186/s12931-015-0249-8

**Published:** 2015-08-18

**Authors:** Paul M. O’Byrne, Tony D’Urzo, Ekkehard Beck, Matjaž Fležar, Martina Gahlemann, Lorna Hart, Zuzana Blahova, Robert Toorawa, Kai-Michael Beeh

**Affiliations:** Firestone Institute for Respiratory Health, and Department of Medicine, McMaster University Medical Centre, 1280 Main Street West, Room 3 W10, Hamilton, ON L8S 4 K1 Canada; Department of Family and Community Medicine, University of Toronto, Toronto, ON Canada; Institut für Gesundheitsförderung GmbH, Rüdersdorf, Germany; Hospital Golnik, Clinical Department of Pulmonology and Allergy, Golnik, Slovenia; Boehringer Ingelheim (Schweiz) GmbH, Basel, Switzerland; Boehringer Ingelheim Canada Ltd, Burlington, ON Canada; Boehringer Ingelheim RCV GmbH & Co. KG, Vienna, Austria; Boehringer Ingelheim Ltd, Bracknell, UK; insaf Respiratory Research Institute, Wiesbaden, Germany

**Keywords:** Olodaterol, Asthma, Long-acting β_2_-agonist, Dose-finding

## Abstract

**Background:**

Olodaterol is a novel, inhaled long-acting β_2_-agonist (LABA) with >24-hour duration of action investigated in asthma and chronic obstructive pulmonary disease.

**Methods:**

Two multicentre studies examined the efficacy and safety of 4 weeks’ once-daily (QD) olodaterol (2, 5, 10 and 20 μg, with background inhaled corticosteroids) in patients with asthma. One randomised, double-blind, parallel-group study (1222.6; 296 patients) administered treatment in the morning. Pulmonary function tests (PFTs) were performed pre-dose (trough) and ≤3 hours post-dose (weeks 1 and 2), and ≤6 hours post-dose after 4 weeks; primary end point was trough forced expiratory volume in 1 second (FEV_1_) response (change from baseline mean FEV_1_) after 4 weeks. A second randomised, double-blind, placebo- and active-controlled (formoterol 12 μg twice-daily) incomplete-block crossover study (1222.27; 198 patients) administered QD treatments in the evening. PFTs were performed over a 24-hour dosing interval after 4 weeks; primary end point was FEV_1_ area under the curve from 0–24 hours (AUC_0–24_) response (change from study baseline [mean FEV_1_] after 4 weeks).

**Results:**

Study 1222.6 showed a statistically significant increase in trough FEV_1_ response with olodaterol 20 μg (0.147 L; 95 % confidence interval [CI]: 0.059, 0.234; *p* = 0.001) versus placebo, with more limited efficacy and no evidence of dose response compared to placebo across the other olodaterol doses (2, 5 and 10 μg). Study 1222.27 demonstrated increases in FEV_1_ AUC_0–24_ responses at 4 weeks with all active treatments (*p* < 0.0001); adjusted mean (95 % CI) differences from placebo were 0.140 (0.097, 0.182), 0.182 (0.140, 0.224), 0.205 (0.163, 0.248) and 0.229 (0.186, 0.272) L for olodaterol 2, 5, 10 and 20 μg, respectively, and 0.169 (0.126, 0.211) for formoterol, providing evidence of increased efficacy with higher olodaterol dose. Olodaterol was generally well tolerated, with a few events associated with known sympathomimetic effects, mainly with 20 μg.

**Conclusions:**

The LABA olodaterol has >24-hour duration of action. In patients with asthma, evidence of bronchodilator efficacy was demonstrated with statistically and clinically significant improvements in the primary end point of trough FEV_1_ response measured in clinics over placebo for the highest administered dose of 20 μg in Study 1222.6, and statistically and clinically significant improvements versus placebo in FEV_1_ AUC_0–24_ responses at 4 weeks for all doses tested in Study 1222.27, which also exhibited a dose response. Bronchodilator efficacy was seen over placebo for all olodaterol doses for morning and evening peak expiratory flow in both studies. All doses were well tolerated.

**Trial registrations:**

NCT00467740 (1222.6) and NCT01013753 (1222.27).

**Electronic supplementary material:**

The online version of this article (doi:10.1186/s12931-015-0249-8) contains supplementary material, which is available to authorized users.

## Background

The use of inhaled long-acting β_2_-agonists (LABAs) is recommended in asthma guidelines as add-on to inhaled corticosteroids (ICS) and has been shown to improve lung function and reduce symptoms and future risk of severe exacerbations [1–3]. Twice-daily (BID) LABAs such as formoterol and salmeterol are well-established controllers in asthma as add-on to ICS [2] and fixed-dose combination products have been available for a number of years. The development of once-daily (QD) LABAs for the treatment of asthma may simplify the dosing strategy and potentially improve adherence and outcomes [4–6].

Olodaterol is a novel LABA with 24-hour bronchodilatory activity and is characterised by high β_2_ selectivity and almost full agonist activity at β_2_ adrenoreceptors [7, 8]. Initial single-dose studies in asthma and chronic obstructive pulmonary disease (COPD) have provided clinical evidence of ≥24-hour activity. In patients with asthma, olodaterol was evaluated in a single-dose study that showed significant protection against methacholine bronchoconstriction compared to placebo for ≤32 hours [9]. In COPD, a Phase II, clinical, single-dose study showed that QD olodaterol effectively maintained bronchodilation over 24 hours [10–12]. Olodaterol has recently been approved at a dose of 5 μg QD for use as maintenance treatment for patients with COPD in Europe and the USA, supported by data from the Phase III olodaterol clinical trial programme that established the long-term efficacy of QD olodaterol for lung-function improvement [13–16].

To further assess the optimum dose of olodaterol in patients with asthma, two dose-finding studies with different designs were conducted sequentially. Both studies aimed to determine the optimum dose of olodaterol inhalation solution delivered by the Respimat® inhaler QD for 4 weeks in patients with asthma. The first study used a parallel-treatment-group design and the second was conducted using an incomplete-block crossover design. Both studies were randomised and double blind. Data from these studies have previously been presented at the American Thoracic Society Annual Meeting [17, 18].

## Methods

### Study design

**Fig. 1:**
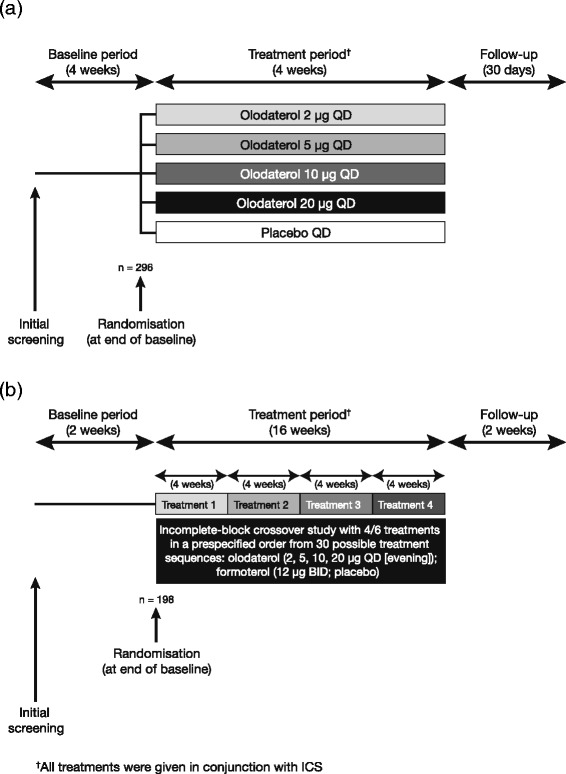
Designs of studies: (**a**) 1222.6 and (**b**) 1222.27. QD: once daily; BID: twice daily; ICS: inhaled corticosteroid

Study 1222.6 was a multicentre, randomised, double-blind, placebo-controlled, parallel-group study to assess the efficacy and safety of 4 weeks of treatment with orally inhaled olodaterol 2, 5, 10 and 20 μg delivered via Respimat® device QD (in the morning) in patients with asthma to determine the optimum dose and confirm 24-hour bronchodilation with this posology (Fig. 1a).

Study 1222.27 was a multicentre, randomised, double-blind, double-dummy, active- and placebo-controlled, incomplete-block crossover study to determine the efficacy and safety of four different doses of olodaterol (2, 5, 10 and 20 μg) delivered by the Respimat® device QD in the evening versus BID formoterol (12 μg) delivered by the Aerolizer® inhaler and matching placebo for 4 weeks in patients with asthma (Fig. 1b). The 16-week treatment phase comprised four 4-week treatment periods without intervening washout periods, with efficacy end points being assessed after 4 weeks of each treatment at study centre visits.

For Study 1222.6, it was estimated that a sample size of 60 patients per treatment group was required to detect with ≥90 % power a difference of 0.15 L from placebo in the primary end point (trough forced expiratory volume in 1 second [FEV_1_] response) at the 5 % significance level.

For Study 1222.27, a sample size of approximately 190 patients was planned to detect a difference from placebo of 0.1 L with 95 % power for a single treatment comparison (assuming a standard deviation for the paired differences of 0.25 L), allowing for the loss of efficiency caused by the incomplete-block crossover design, for patient attrition and in order to achieve a balanced design.

Further details of study designs, assessments performed, key inclusion and exclusion criteria, and statistical methodologies are detailed in Additional file 1: Tables S1 and S2.

Both studies were performed in accordance with the principles laid down by the Declaration of Helsinki, International Committee on Harmonisation, Good Clinical Practice Guidelines, European Medical Device Directive and local regulations. Prior to initiation of the studies, the protocols were approved by the ethics research boards of the respective institutions and written, informed consent was obtained from all patients. Both studies were registered with ClinicalTrials.gov (Study 1222.6: NCT00467740; NCT01013753).

### End points

The primary end point for Study 1222.6 was trough FEV_1_ response (response being defined as change from study baseline mean FEV_1_) at 4 weeks. Secondary and other end points included FEV_1_ area under the curve from 0–3 hours (AUC_0–3_) response (response defined as change from study baseline FEV_1_) after the first dose and after 1, 2 and 4 weeks of treatment, weekly mean pre-dose morning peak expiratory flow (PEF) response (patient diaries), weekly mean evening PEF response, total score on the Asthma Control Questionnaire (ACQ) and safety.

The primary end point for Study 1222.27 was FEV_1_ area under the curve from 0–24 hours (AUC_0–24_) response (defined as change from study baseline mean FEV_1_) after 4 weeks of treatment. Secondary and other end points included FEV_1_ at individual time points over 24 hours post-dose, trough and peak FEV_1_ responses at the end of each 4-week treatment period, weekly pre-dose PEF (from the patient diaries), control of asthma as assessed by total score on the ACQ and quality of life as assessed by the standardised version of the Asthma Quality of Life Questionnaire (AQLQ[S]) at the end of the treatment period.

For both studies, pulmonary function tests (PFTs) were conducted at screening and baseline. In Study 1222.6 (parallel-group design), PFTs were conducted at 1 hour and at 10 min before the morning dose and 30 min, 1, 2 and 3 h post-dosing at the start of randomised treatment and after 1, 2 and 4 weeks of randomised treatment. This was extended to 4, 5 and 6 h post-dosing at the final treatment visit. PFTs were also conducted at follow-up.

In Study 1222.27 (incomplete-block crossover design), PFTs were performed at screening, at the end of the 2-week baseline period, after 4 weeks of each randomised treatment and at follow-up. At the baseline visit, two PFTs were performed at 1 h and at 10 min prior to the first evening dose of study medication. At the end of each 4-week treatment period, PFTs were performed at 1 h and at 10 min before the evening dose and at defined time points within 24 h post-dosing (30 min, 1, 2, 3, 4 h, 11 h 50 min, 12 h 30 min, 13, 14, 15, 16, 18, 20, 22, 23 h and 23 h 50 min post-dose). A single PFT was performed at the follow-up visit.

## Results

**Fig. 2:**
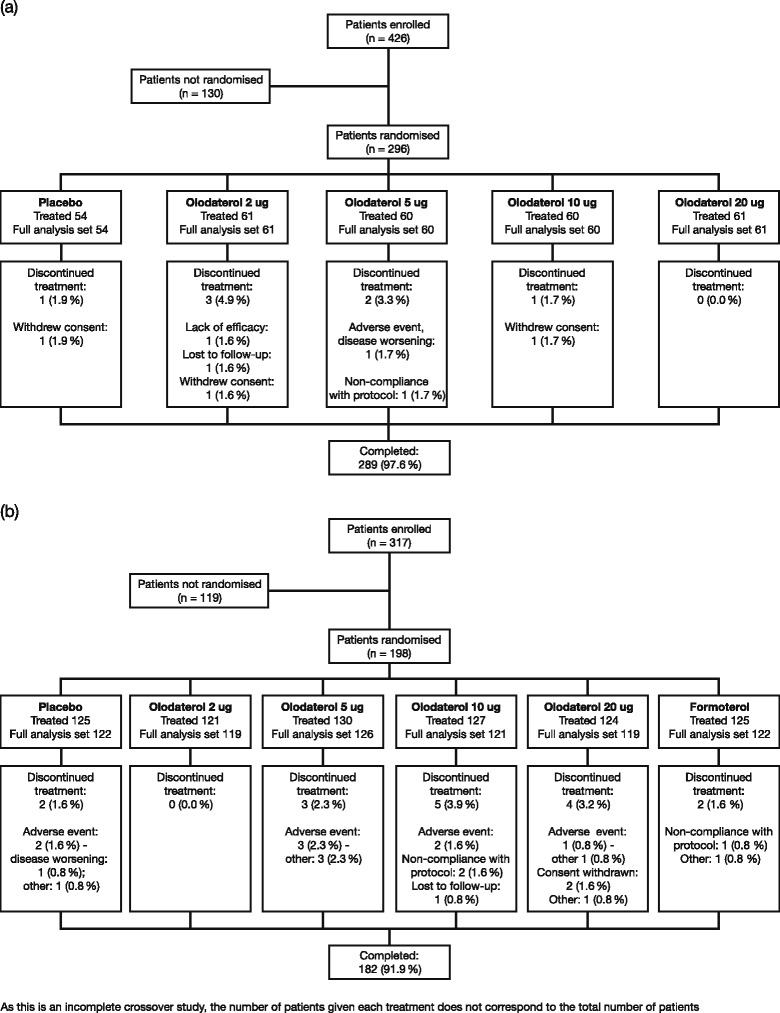
Patient disposition in studies: (**a**) 1222.6 and (**b**) 1222.27

In Study 1222.6, a total of 296 patients were randomised to treatment at 37 centres and 289 patients completed the study (Fig. 2a), while in Study 1222.27, 198 patients were randomised at 27 centres and 182 patients completed the study (Fig. 2b). Across the studies, demographic characteristics were generally similar (Tables 1 and 2). Mean ages in Study 1222.6 ranged between 43.6 and 46.3 years across the treatment groups, while in Study 1222.27 the mean age was 45.0 years; across the studies there were slightly more women than men. The majority of patients were never smokers.

**Table 1:** Baseline patient demographic details in Study 1222.6

	Placebo (n = 54)	Olodaterol 2 μg (n = 61)	Olodaterol 5 μg (n = 60)	Olodaterol 10 μg (n = 60)	Olodaterol 20 μg (n = 61)
Age, mean (SD), years	43.6 (14.1)	45.4 (15.1)	46.2 (13.0)	46.3 (14.5)	44.6 (13.0)
Male, n (%)	20 (37.0)	22 (36.1)	30 (50.0)	26 (43.3)	28 (45.9)
Asthma diagnosis, mean (SD), years	23.7 (13.3)	19.8 (13.8)	23.3 (15.6)	22.5 (15.5)	22.9 (16.2)
Body mass index, mean (SD), kg/m^2^	28.6 (7.2)	27.0 (6.1)	28.8 (6.85)	26.5 (5.2)	27.3 (5.81)
Smoking status, n (%)					
Current smoker	3 (5.6)	7 (11.5)	2 (3.3)	2 (3.3)	2 (3.3)
Ex-smoker	10 (18.5)	16 (26.2)	10 (16.7)	14 (23.3)	15 (24.6)
Never smoker	41 (75.9)	38 (62.3)	48 (80.0)	44 (73.3)	44 (72.1)
Smoking history, mean (SD), pack-years^a^	3.6 (2.4)	4.8 (2.7)	5.5 (2.8)	5.9 (2.7)	3.4 (2.6)
Pre-bronchodilator FEV_1_, mean (SD), L	2.30 (0.62)	2.31 (0.74)	2.39 (0.61)	2.32 (0.64)	2.37 (0.60)
Post-bronchodilator FEV_1_, mean (SD), L	2.72 (0.70)	2.76 (0.91)	2.79 (0.70)	2.74 (0.70)	2.82 (0.69)
Change in FEV_1_ from pre-bronchodilator (SD), L	0.43 (0.15)	0.44 (0.23)	0.41 (0.13)	0.42 (0.16)	0.44 (0.15)
Post-bronchodilator FEV_1_:FVC ratio, mean (SD), %	74.3 (7.4)	75.1 (9.5)	74.2 (7.2)	73.6 (8.5)	74.3 (7.4)

^a^Based on n = 13 for placebo, 23 for 2 μg, 12 for 5 μg, 16 for 10 μg, and 17 for 20 μg (*i.e*. only current and ex-smokers were included in this analysis)

SD: standard deviation; FEV_1_: forced expiratory volume in 1 s; FVC: forced vital capacity

**Table 2:** Baseline patient demographic details in Study 1222.27

	Total (n = 198)
Age, mean (SD), years	45.0 (11.8)
Male, n (%)	86 (43.4)
Asthma diagnosis, mean (SD), years	20.4 (13.2)
Body mass index, mean (SD), kg/m^2^	27.9 (5.3)
Smoking status, n (%)	
Current smoker	1 (0.5)
Ex-smoker	51 (25.8)
Never smoker	146 (73.7)
Smoking history, mean (SD), pack-years^a^	5.4 (2.7)
Pre-bronchodilator FEV_1_, mean (SD), L	2.37 (0.63)
Post-bronchodilator FEV_1_, mean (SD), L	2.92 (0.79)
Change in FEV_1_ from pre-bronchodilator (SD), L	0.55 (0.30)
Post-bronchodilator FEV_1_:FVC ratio, mean (SD), %	71.9 (8.8)

^a^Based on n = 52 (*i.e*. only current and ex-smokers were included in this analysis)

SD: standard deviation; FEV_1_: forced expiratory volume in 1 s; FVC: forced vital capacity

**Fig. 3:**
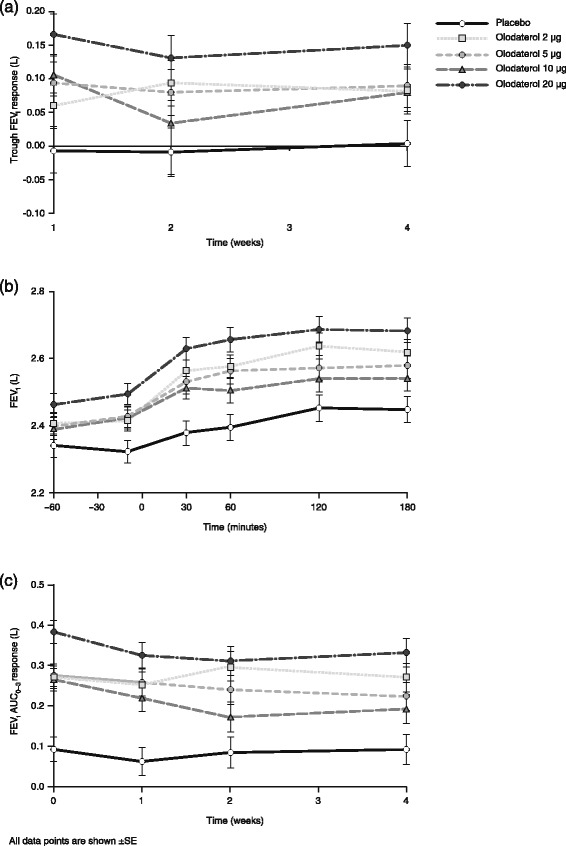
FEV_1_ assessments in Study 1222.6. (**a**) Trough FEV_1_ response weeks 1–4 from baseline (±SE), (**b**) FEV_1_ profile over time at week 4 from baseline (±SE) and (**c**) FEV_1_ AUC_0–3_ response weeks 1–4 from baseline (±SE). FEV_1_: forced expiratory volume in 1 s; SE: standard error; AUC_0–3_: area under the curve from 0–3 hours

In Study 1222.6, for the primary end point of trough FEV_1_ response after 4 weeks of treatment, there was a similar degree of improvement compared with placebo for the 2, 5 and 10 μg doses of olodaterol: mean (95 % confidence interval [CI]) difference of 0.080 (−0.008, 0.167) for the 2 μg olodaterol treatment group, 0.086 (−0.003, 0.174) for the 5 μg treatment group and 0.076 (−0.012, 0.164) for the 10 μg group. The differences between the 20 μg dose and placebo were largest and statistically significant: mean (95 % CI) 0.147 (0.059, 0.234; p = 0.0011) (Fig. 3a and Additional file 1: Table S3). There was no evidence of a dose–response relationship across the 2, 5 and 10 μg doses of olodaterol (Additional file 1: Table S3), with similar results for trough FEV_1_ response observed at weeks 1 and 2 (Fig. 3a).

Figure 3b shows FEV_1_ at individual time points up to 3 h post-dose at week 4 in Study 1222.6; with olodaterol 20 μg these were statistically significant (*p* < 0.001) at all time points. Values for FEV_1_ AUC_0–3_ response were also statistically significantly (*p* < 0.05) higher in each of the olodaterol treatment groups compared to placebo at weeks 1, 2 and 4 (Fig. 3c). For both FEV_1_ over time at week 4 and FEV_1_ AUC_0–3_ at weeks 1, 2 and 4, the same pattern was observed as for the primary end point, with little separation between the 2, 5 and 10 μg doses.

A different pattern was observed for other secondary end points, with some evidence of a dose–response relationship apparent for the mean change in weekly morning pre-dose PEF from baseline (Table 3). Similar results were apparent for the evening PEF response (Table 3). After 4 weeks of treatment, mean total ACQ scores also decreased from baseline in each of the active treatment groups, although the changes compared with placebo were only statistically significant (*p* < 0.05) in the olodaterol 10 and 20 μg dose groups. The mean (95 % CI) differences compared to placebo were -0.142 with 2 μg (−0.368, 0.083; *p* = 0.216), −0.197 with 5 μg (−0.422, 0.029; p = 0.087), −0.328 with 10 μg (−0.552, −0.103; *p* = 0.004) and −0.276 with 20 μg (-0.499, −0.052; *p* = 0.016).

**Table 3:** Weekly pre-dose morning and evening PEF response (L/min) after 4 weeks of treatment for Study 1222.6

			Difference from placebo		
	n	Mean (SE)	Mean (SE)	95 % CI	*p* value
**Morning PEF**					
Placebo	54	368.18 (5.71)			
Olodaterol 2 μg	61	384.42 (5.38)	16.24 (7.84)	0.80, 31.68	0.0393
Olodaterol 5 μg	60	396.06 (5.42)	27.88 (7.88)	12.38, 43.38	0.0005
Olodaterol 10 μg	59	404.26 (5.47)	36.07 (7.91)	20.51, 51.63	<0.0001
Olodaterol 20 μg	61	411.13 (5.38)	42.94 (7.85)	27.50, 58.39	<0.0001
**Evening PEF**					
Placebo	54	384.08 (5.59)			
Olodaterol 2 μg	61	407.05 (5.26)	22.97 (7.67)	7.88, 38.06	0.0030
Olodaterol 5 μg	60	408.68 (5.30)	24.60 (7.70)	9.45, 39.75	0.0016
Olodaterol 10 μg	59	420.88 (5.34)	36.81 (7.73)	21.60, 52.02	<0.0001
Olodaterol 20 μg	61	426.58 (5.26)	42.51 (7.68)	27.40, 57.61	<0.0001

PEF: peak expiratory flow; SE: standard error; CI: confidence interval

In Study 1222.27, the mean FEV_1_ AUC_0–24_ response after 4 weeks of treatment was highly statistically significantly different compared to placebo for all doses of olodaterol and formoterol (*p* < 0.0001) (Additional file 1: Table S4), with a clear dose–response relationship in the treatment effect for olodaterol. The adjusted mean (95 % CI) FEV_1_ AUC_0–24_ response differences from placebo after 4 weeks of treatment were 0.140 (0.097, 0.182), 0.182 (0.140, 0.224), 0.205 (0.163, 0.248) and 0.229 (0.186, 0.272) L for 2, 5, 10 and 20 μg olodaterol, respectively; the adjusted mean difference from placebo with formoterol was 0.169 (0.126, 0.211) L.

**Fig. 4:**
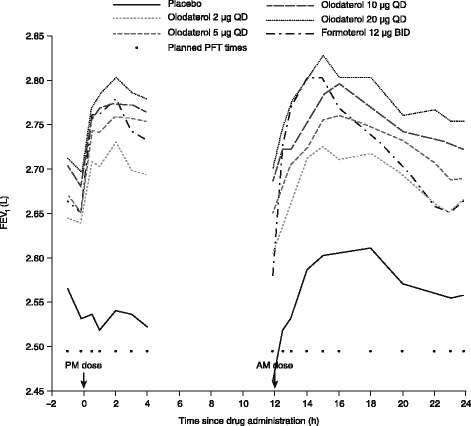
FEV_1_ assessments in Study 1222.27: adjusted mean FEV_1_ trough response at week 4. QD: once daily; BID: twice daily; PFT: pulmonary function test; FEV_1_: forced expiratory volume in 1 s

Differences in adjusted mean FEV_1_ at each individual time point over the 24-h period also demonstrated a clear dose–response relationship (Fig. 4). There were highly statistically significant (*p* < 0.0001) improvements in trough and peak FEV_1_ responses at week 4, again with dose–response relationships apparent with olodaterol (Additional file 1: Table S5).

While there were significant differences in adjusted mean weekly morning PEF and evening PEF (home-measured) at week 4 for all doses of olodaterol and formoterol compared to placebo (*p* < 0.0001), the increases with olodaterol did not show the same dose–response relationship as the in-clinic visit FEV_1_ end points (Additional file 1: Table S6). There were statistically significant reductions in mean total ACQ scores with all active treatments during the study; however, there was no clear dose ordering between the 2, 5 and 10 μg doses. At week 4, the mean (95 % CI) differences compared to placebo were −0.321 (−0.432, −0.210) with the 2 μg dose, −0.293 (−0.403, −0.184) with the 5 μg dose, −0.326 (−0.436, −0.215) with the 10 μg dose and −0.394 (−0.505, −0.282) with the 20 μg dose; all differences were highly statistically significant (*p* < 0.0001). Compared to placebo, the mean (95 % CI) difference with formoterol was −0.346 (−0.457, −0.235; *p* < 0.0001). There were also statistically significant increases in AQLQ(S) score at week 4 with all active treatments compared to placebo (*p* < 0.001). Mean (95 % CI) differences to placebo were 0.289 (0.178, 0.400) with olodaterol 2 μg, 0.209 (0.099, 0.318) with the 5 μg dose, 0.262 (0.151, 0.374), with the 10 μg dose, 0.317 (0.205, 0.429) with the 20 μg dose and 0.315 (0.203, 0.426) with formoterol.

The incidence of adverse events in Study 1222.6 was 37.7 %, 40.0 %, 30.0 %, 39.3 % and 27.8 % in patients receiving olodaterol 2 μg, 5 μg, 10 μg, 20 μg and placebo, respectively (Additional file 1: Table S7). Exacerbation of asthma occurred at a higher incidence in the placebo treatment group (7.4 % compared to 1.7–3.3 % in the olodaterol groups). Only 24 adverse events (total: all treatment groups) were considered drug related, the most common being tremor, headache, dizziness, palpitations and anxiety. However, there was a higher number of patients with drug-related adverse events in the olodaterol 20 μg group compared to the lower-dose groups (16.4 % compared to 3.3–8.3 % in the other groups), the most frequent being tremor.

One patient receiving olodaterol 5 μg was withdrawn from Study 1222.6 due to an adverse event of premature ventricular contractions. Two patients experienced at least one serious adverse event during the study: one in the 10 μg group experienced pneumonia and one in the 20 μg group experienced dizziness, palpitations, hyperhidrosis and chest pain, which were considered drug related. None of the serious adverse events were fatal or life-threatening.

More female patients in the olodaterol 20 μg group experienced drug-related adverse events (seven patients; 21.2 %) than in the placebo group (two patients; 5.1 %) or other active treatment groups (one to three patients; 3.3–8.8 %). No such difference was seen for male patients.

Changes in vital signs, electrocardiogram and laboratory parameters in line with known systemic sympathomimetic effects were observed in Study 1222.6 with olodaterol doses ≥10 μg, with increased heart rate, shortened uncorrected QT interval, increased QT (Bazett corrected) and T-wave abnormalities. Although there was a statistically significant reduction in mean serum potassium levels in patients with the 20 μg olodaterol dose compared to placebo following treatment on day 1 (4.18 versus 4.38 mmol/L; p = 0.0006), no statistically significant treatment differences between placebo and any active treatment dose groups were observed at other time points during the study. Two electrocardiogram results were reported as adverse events: one patient in the placebo group with an atrioventricular block and one in the 5 μg group with ventricular extrasystoles.

The overall incidence of adverse events in Study 1222.27 was 9.9 %, 13.8 %, 15.7 %, 12.9 %, 6.4 % and 4.8 % in patients receiving olodaterol 2 μg, 5 μg, 10 μg, 20 μg, formoterol and placebo, respectively. The differences in overall adverse event incidences between treatments could not be explained by more frequent occurrences of any particular preferred term. Only three adverse events were reported by >1 % of patients on any treatment: nasopharyngitis, dyspnoea and headache (Additional file 1: Table S8). Two patients experienced serious adverse events: one patient being treated with 10 μg experienced appendicitis and one patient in the post-treatment period (last treatment olodaterol 2 μg) had a cerebral infarction. Both patients recovered (the patient with cerebral infarction recovered with sequelae) and neither serious adverse event was considered drug related.

Eight adverse events were considered related to study drug in Study 1222.27: four with olodaterol 5 μg (candidiasis, headache, palpitations and muscle spasms), two with olodaterol 10 μg (palpitations and muscle spasms) and one each with olodaterol 2 μg (insomnia) and placebo (metrorrhagia). No drug-related adverse events were reported by patients being treated with 20 μg olodaterol or formoterol.

With the exception of olodaterol 20 μg, there was generally a greater percentage of female patients reporting at least one adverse event with olodaterol treatment: 11.4 % (2 μg), 18.3 % (10 μg), 22.2 % (15 μg) and 12.7 % (20 μg) with olodaterol compared to 4.1 % with placebo and 5.8 % with formoterol. This is in comparison to 7.8 % (2 μg), 12.5 % (10 μg), 3.4 % (15 μg) and 13.2 % (20 μg) of male patients following olodaterol treatment and 5.8 % and 7.1 % following treatment with placebo or formoterol, respectively.

Analysis of the electrocardiogram results showed that, compared to placebo, there was a slight increase (mean change from baseline: 4.23 ms at 1 h) in the mean Fridericia-corrected QT interval with 20 μg olodaterol. There were statistically significant (*p* < 0.03) reductions in mean blood potassium concentration seen 1 h post-dose: ratios of geometric mean olodaterol:placebo (95 % CI) were 0.979 (0.962, 0.997) with 5 μg, 0.977 (0.960, 0.995) with 10 μg and 0.980 (0.962, 0.998) with 20 μg olodaterol; however, these changes were not considered to be clinically relevant.

One electrocardiogram result was reported as an adverse event: one patient with an atrioventricular block while receiving olodaterol 20 μg and formoterol.

## Discussion

These studies have demonstrated that olodaterol is an inhaled LABA with a duration of effect lasting >24 h in patients with asthma when administered in addition to ICS over 4 weeks, as measured by improvements over placebo in FEV_1_ and home-measured morning and evening PEF in both studies. Olodaterol also improved symptoms as measured by a reduction in ACQ scores. Both studies demonstrated or indicated dose–response effects of olodaterol to a greater (Study 1222.27) or lesser (Study 1222.6) degree across the 2, 5, 10 and 20 μg doses used. The outcome variables that indicated olodaterol dose response in the two studies varied, being indicated only by secondary end points of weekly morning and evening PEF in Study 1222.6, and by the primary end point of FEV_1_ AUC_0–24_ response and secondary end points of trough, peak and individual time point FEV_1_ responses in Study 1222.27 (as well as other end points studied in 1222.27).

Potential reasons for the differences in the outcome variables demonstrating dose response between the studies could include the populations and/or the designs used in the two studies. However, the study populations were similar with regards to patient demographics; all patients were taking ICS and both populations demonstrated a dose response to olodaterol in some outcome variables. Therefore, it is unlikely that an important difference in patient characteristics is an adequate explanation. By contrast, the study designs used were very different. Study 1222.6 used a parallel-group design and Study 1222.27 an incomplete-block crossover design, with each patient receiving four out of a possible six treatments and acting as their own controls for the dose–response evaluations and an active comparator with formoterol. Another important difference in the design of the studies was the time of administration of olodaterol: in the morning in Study 1222.6 and in the evening in Study 1222.27.

Another study that examined the dose–response characteristics of olodaterol was also a crossover design and included patients with milder asthma, not taking ICS. The primary outcome variable was change in methacholine provocative concentration causing a 20 % fall in FEV_1_ (PC_20_). Olodaterol demonstrated a clear dose response for the changes in methacholine PC_20_ [9].

There are challenges in conducting β_2_-agonist dose–response studies in asthma. Studies examining the dose response of β_2_-agonists using bronchoprovocation models in asthma are inherently easier to conduct, as they involve mild, stable patients with close to normal baseline FEV_1_ values. When indices of bronchodilation are used to measure effect, the patients need to have baseline airflow obstruction in order to demonstrate a response and in most countries such patients would be treated with ICS. These patients may also be previously treated with LABA and washed out from this medication (for a minimum of 2 weeks in Study 1222.6 and 48 h in Study 1222.27).

Other LABAs with a duration of action of >24 h have been studied in patients with asthma. Beeh *et al*. demonstrated that indacaterol had a duration of bronchodilation of 24 h for the two highest doses but did not demonstrate a significant dose response for FEV_1_ across four doses administered [19]. Another study that included patients taking ICS and which evaluated four doses of indacaterol treatment for 7 days also did not demonstrate a dose response when FEV_1_ was measured [20]. The dose–response characteristics of vilanterol, another LABA, have been evaluated in patients with asthma taking ICS measured by treating with five different doses for 28 days and measuring both FEV_1_ and PEF [21]. Once again, while prolonged bronchodilation was observed for the three highest doses used, a significant dose response was not seen for either outcome variable. These studies demonstrate the challenges in documenting a clear dose response for a LABA seen in a primary end-point measure (as in Study 1222.27), although evidence for a dose response may be apparent in secondary end-point measures (as in Study 1222.6).

The safety profile of olodaterol was as expected for all β_2_-agonists, with tremor and changes in serum potassium and the QT interval in the electrocardiogram at the highest dose. There was no evidence of an increase of severe adverse events with olodaterol when compared to placebo in Study 1222.6 or formoterol in Study 1222.27.

These studies did have some limitations in that neither study was adequately powered to identify differences between the different doses of active treatments (both studies were powered for comparisons between olodaterol and placebo), though this is not unusual for Phase II dose-finding studies. When taken together, the weight of the evidence from several olodaterol trials in asthma indicates a relevant dose–response relationship between a total daily dose of 5 and 10 μg olodaterol. In addition to the results from these present studies, further evidence has been provided by a study examining the 24-h FEV_1_ time profile after 3 weeks of treatment with QD or BID regimens of olodaterol (at the same total daily dose) versus placebo (NCT01311661; 1222.29). Dose responses, although not the primary objective of the studies, were consistently observed for a total daily dose of 5 versus 10 μg olodaterol (Beeh et al., manuscript in preparation).

In conclusion, treatment with olodaterol QD for 4 weeks was well tolerated at all doses and no new safety concerns were identified, although some sympathomimetic effects were observed, mainly at the 20 μg dose. Parallel, as well as incomplete-block crossover, designs may be suitable for Phase II dose-ranging studies and more than one study may be necessary to strengthen the evidence for a dose response.

## Additional file

Additional file 1: Table S1. Study methodology. **Table S2.** Key inclusion and exclusion criteria. **Table S3.** Adjusted mean (95 % CI) FEV_1_ trough and peak responses compared to placebo after 4 weeks of treatment in Study 1222.6. **Table S4.** Adjusted mean (95 % CI) FEV_1_ AUC_0–24_ responses compared to placebo after 4 weeks of treatment in Study 1222.27. **Table S5.** Adjusted mean (95 % CI) FEV_1_ responses compared to placebo after 4 weeks of treatment in Study 1222.27. **Table S6.** Adjusted mean (95 % CI) morning and evening PEF compared to placebo after 4 weeks of treatment in Study 1222.27. **Table S7.** Adverse events reported in Study 1222.6 occurring in more than one patient in any treatment group. **Table S8.** Adverse events reported in Study 1222.27 with an incidence of >1 % on any treatment on any system class or individual adverse event.

## Abbreviations

ACQ: Asthma Control Questionnaire
AQLQ(S): Asthma Quality of Life Questionnaire, standardised version
AUC_0–3_: Area under the curve from 0–3 hours
AUC_0–24_: Area under the curve from 0–24 hours
BID: Twice daily
CI: Confidence interval
FEV_1_: Forced expiratory volume in 1 second
ICS: Inhaled corticosteroids
LABA: Long-acting β_2_-agonist
PEF: Peak expiratory flow
PFT: Pulmonary function test
QD: Once daily
