# Supplementary material for: Dose-finding evaluation of once-daily treatment with olodaterol, a novel long-acting β2-agonist, in patients with asthma: results of a parallel-group study and a crossover study
Source: Respir Res. 2015 Aug 18;16(1):97. doi: 10.1186/s12931-015-0249-8 (PMC4539885; doi:10.1186/s12931-015-0249-8)
Supplement: Additional file 1: Table S1. — Study methodology. Table S2. Key inclusion and exclusion criteria. Table S3. Adjusted mean (95 % CI) FEV1 trough and peak responses compared to placebo after 4 weeks of treatment in Study 1222.6. Table S4. Adjusted mean (95 % CI) FEV1 AUC0–24 responses compared to placebo after 4 weeks of treatment in Study 1222.27. Table S5. Adjusted mean (95 % CI) FEV1 responses compared to placebo after 4 weeks of treatment in Study 1222.27. Table S6. Adjusted mean (95 % CI) morning and evening PEF compared to placebo after 4 weeks of treatment in Study 1222.27. Table S7. Adverse events reported in Study 1222.6 occurring in more than one patient in any treatment group. Table S8. Adverse events reported in Study 1222.27 with an incidence of >1 % on any treatment on any system class or individual adverse event. [file 12931_2015_249_MOESM1_ESM.doc]

**Table S1 Study methodology**

| **Study design**  Study 1222.6 was a multicentre, randomised, double-blind, placebo-controlled, parallel-group study to assess the efficacy and safety of 4 weeks of QD (AM) treatment with orally inhaled olodaterol 2, 5, 10 and 20 µg in patients with asthma to determine the optimum dose of olodaterol and confirm 24-hour bronchodilation with this posology. Following an initial screening phase, patients entered a 4-week baseline period. Patients were then randomised into the 4-week, double-blind treatment phase in which they received one of 2, 5, 10 or 20 µg olodaterol or placebo QD via a Respimat® inhaler. Trial medication was administered between 07:00 and 10:00 in the morning each day as two actuations via a Respimat® inhaler. All patients were required to take low to moderate doses of ICS throughout the study (and for 12 weeks prior to screening) and rescue medication (salbutamol 100 µg per actuation) was allowed. Study centre visits during treatment occurred for screening, day 1 and at weeks 1, 2 and 4. Patients were contacted by telephone after 3 weeks of treatment to assess clinical status, adverse events and changes in concomitant medication. Patients were followed-up 30 days after completion of the randomised treatment period.  Study 1222.27 was a multicentre, randomised, double-blind, double-dummy, active- and placebo-controlled, incomplete-block crossover study to determine the efficacy and safety of four different doses of olodaterol (2, 5, 10 and 20 µg) delivered by the Respimat® device QD in the evening versus BID formoterol (12 µg) delivered by the Aerolizer® inhaler and matching placebo for 4 weeks in patients with asthma. All patients were required to take stable low to moderate doses of ICS throughout the study. After an initial screening visit, patients entered a 2-week screening period during which they ~~were required to take stable doses of ICS and~~ could also receive salbutamol (100 μg per actuation) as rescue medication. Patients were then randomised to one of 30 sequences comprising four out of the six possible treatments in a pre-specified order, according to an incomplete-block crossover design. The possible treatments in each sequence were the four doses of olodaterol QD in the evening via the Respimat® inhaler, formoterol 12 μg BID via the Aerolizer® inhaler and placebo via the Respimat® and Aerolizer® inhalers (as olodaterol and formoterol require different modes of administration, a double-dummy method was used to ensure blinding). Each morning between 06:00 and 08:00, patients self-administered formoterol (or corresponding placebo) as one actuation of the Aerolizer® inhaler. Each evening between 18:00 and 20:00, patients received olodaterol (or corresponding placebo) as two actuations of the Respimat® inhaler followed by formoterol (or corresponding placebo) as one actuation of the Aerolizer® inhaler. All patients continued to receive stable doses of ICS as background treatment as individually prescribed in the morning/evening after inhalation of study treatment.  The 16-week treatment phase comprised four 4-week treatment periods without intervening washout periods, with efficacy end points being assessed after 4 weeks of each treatment at study centre visits. For safety monitoring and to ensure correct medication administration, patients were contacted by telephone between visits. Patients were required to return for a follow-up visit 2 weeks after completion of randomised treatment.  Key inclusion and exclusion criteria are listed in Table S2. For both studies, patients had to have taken ICS for ≥12 weeks before screening to be eligible for participation in the trial and had to have received a stable dose of a medium-/high-dose ICS or a low- to high-dose ICS in combination with a LABA (for Study 1222.27) or a low/moderate dose of ICS (for Study 1222.6) for 6 weeks before screening. Low to high doses of glucosteroids were defined according to the GINA criteria [1]. ICS medications included beclomatasone, budesonide, fluticasone, triamcinolone, ciclesonide and mometasone.  **Procedures**  For both studies, PFTs were conducted at screening and baseline. All spirometers and their usage had to meet American Thoracic Society/European Respiratory Society criteria [2]. In Study 1222.6 (parallel-group design), PFTs were conducted at 1 hour and at 10 minutes before the morning dose and 30 minutes, 1, 2 and 3 hours post-dosing at the start of randomised treatment and after 1, 2 and 4 weeks of randomised treatment. This was extended to 4, 5 and 6 hours post-dosing at the final treatment visit. PFTs were also conducted at follow-up. In addition, BID peak-flow measurements were recorded by the patient throughout the study, including during the 4-week baseline period, the 4-week treatment period (with the exception of the clinic visits) and the 30-day post-treatment period. Morning measurements were performed immediately on rising and prior to taking trial medication (or rescue medication), and evening measurements were taken at bedtime. The best of three readings for each measurement were recorded. Patients used an electronic diary (Asthma Monitor® AM2+ [VIASYS, Hoechberg, Germany]) to record the results of the BID PEF, study medication use and rescue salbutamol (albuterol) use (data not reported).  In Study 1222.27 (incomplete-block crossover design), PFTs were performed at screening, at the end of the 2-week baseline period, after 4 weeks of each randomised treatment and at follow-up. At the baseline visit, two PFTs were performed at 1 hour and at 10 minutes prior to the first evening dose of study medication. At the end of each 4-week treatment period, PFTs were performed at 1 hour and at 10 minutes before the evening dose and at defined time points within 24 hours post-dosing (30 minutes, 1, 2, 3, 4 hours, 11 hours 50 minutes, 12 hours 30 minutes, 13, 14, 15, 16, 18, 20, 22, 23 hours and 23 hours 50 minutes post-dose). A single PFT was performed at the follow-up visit.  Patients used an electronic diary (Asthma Monitor® AM2+) BID (morning and evening) to record asthma symptoms, nocturnal awakenings, rescue medication and ICS use, and adherence to study medication (during the treatment periods), and to measure PEF (morning and evening) and FEV_1_. Morning PEF and FEV_1_ measurements were performed immediately on rising and both morning and evening recordings were made prior to administration of the trial medication, maintenance ICS or any other pulmonary medication. The highest PEF and FEV_1_ measurements recorded by the patient out of three acceptable blows were used for evaluation.  In Study 1222.6, the ACQ was completed at baseline and after 4 weeks of randomised treatment [3]. In Study 1222.27, the AQLQ(S) [4] was completed at baseline, after each 4-week treatment period and at the follow-up visit. In both studies, pulse rate and blood pressure were taken in the clinic at 1 hour and at 10 minutes prior to dosing and 30 minutes, 1, 2 and 3 hours post-dosing (and 6 hours post-dose in Study 1222.6) after 4 weeks of each treatment, and in Study 1222.6 at 1 and 2 weeks. Clinical laboratory tests were performed during screening and at the last visit on treatment for both studies and at weeks 1 and 2 for Study 1222.6. Abbreviated laboratory tests to measure potassium levels were performed in Study 1222.27 pre-dose and at 1 and 3 hours post-dose at weeks 4, 8, 12 and 16. Physical examinations were performed during screening and at the last visit on treatment in both studies. 12-lead electrocardiogram was performed at screening and on treatment at 30 minutes pre-dose and up to 3 hours post-dose at weeks 1 and 2 in Study 1222.6 and at the end of each treatment period in Study 1222.27 and at end of treatment. Adverse events and the use of concomitant medications were recorded on the case report form at each visit, medication washout compliance was checked and e-diary data reviewed. Pharmacokinetics were assessed at each clinic visit during the treatment period (results not shown).  **Statistical methodology**  ~~For~~ *Study 1222.6*~~, it was estimated that a sample size of 60 patients per treatment group was required to detect with ≥90% power a difference of 0.15 L from placebo in the primary end point (trough FEV~~_~~1~~_ ~~response) at the 5% significance level.~~  The analysis of the primary end point (trough FEV_1_ response) used a mixed-effects linear model to compare bronchodilation among the five treatment groups after 4 weeks of treatment. Mean changes from baseline to each of the weekly treatment visits were analysed using a restricted maximum-likelihood-based repeated measures approach. Analyses included the fixed effects of treatment, visit and treatment-by-visit interaction, as well as the continuous covariate of baseline measurement and baseline-by-visit interaction. The primary analysis was performed on the full analysis set (i.e. all patients who took at least one dose of study medication with at least one pre-dose and one post-treatment FEV_1_ measurement). For trough FEV_1_ response, each dose was tested separately against placebo with multiplicity controlled by a stepwise procedure.  Secondary efficacy lung-function end points were analysed in a similar manner to the primary end point (i.e. a mixed-effects linear model). Total ACQ scores (i.e. the average of the individual scores) were analysed using analysis of covariance with centre as random effect.  ~~For~~ *Study 1222.27*~~, a sample size of approximately 190 patients was planned to detect a difference from placebo of 0.1 L with 95% power for a single treatment comparison (assuming a standard deviation for the paired differences of 0.25 L), allowing for the loss of efficiency caused by the incomplete-block crossover design, for patient attrition and in order to achieve a balanced design.~~  The analysis of the primary end point (FEV_1_ AUC_0–24_ response) used a mixed model suitable for a crossover study, comprising ‘treatment’, ‘period’ and ‘baseline FEV_1_’ as fixed effects, and ‘patient’ as a random effect. Since the study design was an incomplete-block crossover, and there may additionally have been patients who did not have data for all four crossover periods, each estimate of treatment effect was a weighted average of within-patient and between-patient estimates. The primary analysis was performed on the full analysis set, defined as all patients who received at least one dose of any treatment, who had at least one pre-dose FEV_1_ value at study baseline and at least one post-dose FEV_1_ value at an on-treatment visit. Secondary end points based on spirometry, the ACQ and AQLQ(S) were analysed using a mixed model for a crossover study similar to the primary analysis model, except that the relevant ‘baseline’ was fitted as a continuous fixed effect.  Safety data were mainly presented using descriptive statistics based on the treated set (i.e. all patients who had taken at least one dose of study medication), though log-potassium levels in Study 1222.27 were analysed using a mixed model for a crossover study, similar to the primary analysis but with the relevant ‘baseline’ as a continuous fixed effect. |
| --- |

QD: once daily; ICS: inhaled corticosteroid; BID: twice daily; GINA: Global Initiative for Asthma; PFT: pulmonary function test; PEF: peak expiratory flow; FEV_1_: forced expiratory volume in 1 second; ACQ: Asthma Control Questionnaire; AQLQ(S): Asthma Quality of Life Questionnaire, standardised version; AUC_0–24_: area under the curve from 0–24 hours.

**References**

1. Global Initiative for Asthma. Global strategy for asthma management and prevention. Revised 2006. [http://www.ginasthma.org/local/uploads/files/GINA_Report_072007_1.pdf](http://www.ginasthma.org/local/uploads/files/GINA_Report_072007_1.pdf%5D). Accessed 1 Jun 2015.

2. Miller MR, Hankinson J, Brusasco V, Burgos F, Casaburi R, Coates A et al. Standardisation of spirometry. Eur Respir J. 2005;26:319–38.

3. Juniper EF, O'Byrne PM, Guyatt GH, Ferrie PJ, King DR. Development and validation of a questionnaire to measure asthma control. Eur Respir J. 1999;14:902–7.

4. Juniper EF, Buist AS, Cox FM, Ferrie PJ, King DR. Validation of a standardized version of the Asthma Quality of Life Questionnaire. Chest 1999;115:1265–70.

**Table S2 Key inclusion and exclusion criteria**

| **Study 1222.6** | **Study 1222.27** |
| --- | --- |
| **Inclusion criteria** |  |
| - Patients of either sex, aged ≥18 years with a diagnosis of asthma (based on GINA criteria) - Pre-bronchodilator FEV_1_ of ≥60% to <90% predicted, and an increase in FEV_1_ of ≥12% and 200 mL 15 minutes after receiving 400 µg salbutamol - Use of ICS for the preceding 12 weeks, with a stable low/moderate dose for 6 weeks - Ability to inhale medication in a competent manner from the Respimat® inhaler and from an MDI - Ability to perform PFTs (supervised and unsupervised) and PEF measurements and maintain records during trial period | - Patients of either sex, aged ≥18–70 years, diurnally active, with a ≥3-month diagnosis of asthma (GINA treatment steps 3 or 4), made before the age of 40 years - Pre-bronchodilator FEV_1_ of ≥60% to <90% predicted, and an increase in FEV_1_ of ≥12% and ≥200 mL 15 minutes after receiving 400 µg salbutamol - Use of ICS for the preceding 12 weeks, with a stable dose for 6 weeks: either medium- to high-dose ICS or low- to high-dose ICS + LABA (LABA component not permitted within 48 hours of screening) - Ability to inhale medication in a competent manner from the Respimat® inhaler, the Aerolizer® inhaler and from an MDI - Ability to perform PFTs (supervised and unsupervised) and PEF measurements and maintain records during trial period - Symptomatic as defined by ACQ mean score ≥1.5 - Current non-smokers or ex-smokers (≥1 year) with a cigarette smoking history of <10 pack-years - Willing to continue any pulmonary medication allowed by the trial protocol for the duration of the study |
| **Exclusion criteria** |  |
| - Significant diseases other than asthma (based on investigator’s judgement): history of myocardial infarction in the preceding year, clinically relevant cardiac arrhythmia, cor pulmonale, active tuberculosis, life-threatening pulmonary obstruction, cystic fibrosis, clinically evident bronchiectasis, significant alcohol or drug abuse, previous thoracotomy with pulmonary resection, or malignancy requiring resection, radiotherapy or chemotherapy in the previous 5 years - Abnormal haematology, blood chemistry or urinalysis; patients with serum glutamic oxaloacetic transaminase >80 IU/L, serum glutamic pyruvate transaminase >80 IU/L, bilirubin >1.5 × ULN or creatinine >1.5 × ULN (men)/ 95 µmol/L (women) - Thyrotoxicosis, paroxysmal tachycardia, marked prolongation of QT/QTc interval, risk factors for Torsade de Pointes - Use of LABAs (within 2 weeks of entry), medications that prolong the QT/QTc interval of the ECG, oral β-adrenergics or β-blockers - Inability to comply with pulmonary restrictions prior to randomisation - Patients with a smoking history of >10 pack-years - Patients who had taken an investigational drug within 1 month or 6 half-lives prior to screening - Patients who had previously been randomised in this study or were participating in another study - Known hypersensitivity to β-adrenergics drugs or any component of the Respimat® device - Pregnant or nursing women or women of childbearing potential not using a highly effective method of birth control | - Significant diseases other than asthma (based on investigator’s judgement): history of myocardial infarction in the preceding year, clinically relevant cardiac arrhythmia, cor pulmonale, active tuberculosis, life-threatening pulmonary obstruction, COPD, cystic fibrosis, clinically evident bronchiectasis, significant alcohol or drug abuse, previous thoracotomy with pulmonary resection, or malignancy requiring resection, radiotherapy or chemotherapy in the previous 5 years - Abnormal haematology, blood chemistry or urinalysis; AST >80 IU/L, ALT >80 IU/L, bilirubin or creatinine >1.5 × ULN - Thyrotoxicosis, paroxysmal tachycardia, marked prolongation of QT/QTc interval, risk factors for Torsade de Pointes - Frequent seasonal exacerbations - Asthma exacerbation or respiratory tract infection in the 4 weeks prior to screening or during baseline period - Use of LABAs (within 48 hours of entry), oral or other systemic corticosteroids (within 6 weeks), anti-IgE (within 6 months), medications that prolong the QT/QTc interval of the ECG, oral β-adrenergics (within 6 weeks), β-blockers, methylxanthines and anti-leukotrienes (within 6 weeks), short-acting anticholinergics including fixed-dose anticholinergic/β-adrenergic combinations (within 8 hours), or long-acting anticholinergics (within 6 weeks) - Patients who started immunotherapy within the previous 2 years, not on a stable dose - Inability to comply with pulmonary restrictions prior to randomisation - Patients who had taken an investigational drug within 1 month or 6 half-lives prior to screening - Patients who had previously been randomised in this study or were participating in another study - Patients whose compliance using the AM2+ e-diary device was <80% during baseline period - Known hypersensitivity to β-adrenergics drugs or any component of the Respimat® device - Pregnant or nursing women or women of childbearing potential not using a highly effective method of birth control - Patients who were predominantly night-active (e.g. working night shifts) |

GINA: Global Initiative for Asthma; FEV_1_: forced expiratory volume in 1 second; ICS: inhaled corticosteroid; MDI: metered-dose inhaler; PEF: peak expiratory flow; PFT: pulmonary function test; LABA: long-acting β_2_-agonist; ACQ: Asthma Control Questionnaire; COPD: chronic obstructive pulmonary disease; ULN: upper limit of normal; AST: aspartate aminotransferase; ALT: alanine aminotransferase; QTc: corrected QT; IgE: immunoglobulin E; ECG: electrocardiogram; AM2+: Asthma Monitor-2®.

**Table S3 Adjusted mean (95% CI) FEV_1_ trough and peak responses compared to placebo after 4 weeks of treatment in Study 1222.6**

|  | **n** | **FEV_1_ response, L Adjusted mean difference from placebo** | | |
| --- | --- | --- | --- | --- |
|  |  | **Mean** | **95% CI** | **p value** |
| **Trough FEV_1_** |  |  |  |  |
| Olodaterol 2 µg | 61 | 0.080 | -0.008, 0.167 | 0.0754 |
| Olodaterol 5 µg | 59 | 0.086 | -0.003, 0.174 | 0.0571 |
| Olodaterol 10 µg | 60 | 0.076 | -0.012, 0.164 | 0.0906 |
| Olodaterol 20 µg | 61 | 0.147 | 0.059, 0.234 | 0.0011 |
| **Peak_0–3_ FEV_1_** |  |  |  |  |
| Olodaterol 2 µg | 61 | 0.166 | 0.060, 0.271 | 0.0022 |
| Olodaterol 5 µg | 60 | 0.117 | 0.011, 0.223 | 0.0307 |
| Olodaterol 10 µg | 60 | 0.081 | -0.025, 0.187 | 0.1320 |
| Olodaterol 20 µg | 61 | 0.232 | 0.127, 0.337 | < 0.0001 |

The adjusted mean change in trough FEV_1_ after 4 weeks in the placebo group was 0.004 L. The adjusted mean change in peak_0–3_ FEV_1_ after 4 weeks in the placebo group was 0.198 L.
CI: confidence interval; FEV_1_: forced expiratory volume in 1 second; peak_0–3_ FEV_1_: highest FEV_1_ from 0–3 hours.

**Table S4 Adjusted mean (95% CI) FEV_1_ AUC_0–24_ responses compared to placebo after 4 weeks of treatment in Study 1222.27**

|  | **n** | **FEV_1_ AUC_0–24_ response, L Adjusted mean difference from placebo** | | |
| --- | --- | --- | --- | --- |
|  |  | **Mean** | **95% CI** | **p value** |
| Olodaterol 2 µg | 119 | 0.140 | 0.097, 0.182 | < 0.0001 |
| Olodaterol 5 µg | 126 | 0.182 | 0.140, 0.224 | < 0.0001 |
| Olodaterol 10 µg | 121 | 0.205 | 0.163, 0.248 | < 0.0001 |
| Olodaterol 20 µg | 119 | 0.229 | 0.186, 0.272 | < 0.0001 |
| Formoterol | 122 | 0.169 | 0.126, 0.211 | < 0.0001 |

The adjusted mean change in FEV_1_ AUC_0–24_ after 4 weeks in the placebo group was -0.004 L.
CI: confidence interval; FEV_1_: forced expiratory volume in 1 second; AUC_0–24_: area under the curve from 0–24 hours.

**Table S5 Adjusted mean (95% CI) FEV_1_ responses compared to placebo after 4 weeks of treatment in Study 1222.27**

|  | **n** | **FEV_1_ response, L Adjusted mean difference from placebo** | | |
| --- | --- | --- | --- | --- |
|  |  | **Mean** | **95% CI** | **p value** |
| **Trough FEV_1_** |  |  |  |  |
| Olodaterol 2 µg | 119 | 0.103 | 0.058, 0.148 | < 0.0001 |
| Olodaterol 5 µg | 126 | 0.133 | 0.088, 0.177 | < 0.0001 |
| Olodaterol 10 µg | 121 | 0.169 | 0.124, 0.214 | < 0.0001 |
| Olodaterol 20 µg | 119 | 0.199 | 0.153, 0.244 | < 0.0001 |
| Formoterol | 122 | 0.103 | 0.058, 0.147 | < 0.0001 |
| **Peak FEV_1_** |  |  |  |  |
| Olodaterol 2 µg | 119 | 0.102 | 0.056, 0.148 | < 0.0001 |
| Olodaterol 5 µg | 126 | 0.135 | 0.090, 0.180 | < 0.0001 |
| Olodaterol 10 µg | 121 | 0.161 | 0.116, 0.207 | < 0.0001 |
| Olodaterol 20 µg | 119 | 0.180 | 0.134, 0.226 | < 0.0001 |
| Formoterol | 122 | 0.166 | 0.120, 0.211 | < 0.0001 |

The adjusted mean changes in trough and peak FEV_1_ after 4 weeks in the placebo group were 0.013 and 0.224 L, respectively.
CI: confidence interval; FEV_1_: forced expiratory volume in 1 second.

***Table S6 Adjusted mean (95% CI) morning and evening PEF compared to placebo after 4 weeks of treatment in Study 1222.27***

| **Morning PEF, L/min** | **n** |  | **Adjusted mean difference from placebo** | | |
| --- | --- | --- | --- | --- | --- |
|  |  | **Mean (SE)** | **Mean (SE)** | **95% CI** | **p value** |
| Placebo | 122 | 362.71 (4.72) |  |  |  |
| Olodaterol 2 µg | 119 | 383.13 (4.75) | 20.42 (4.09) | 12.40, 28.45 | < 0.0001 |
| Olodaterol 5 µg | 126 | 390.81 (4.69) | 28.11 (4.02) | 20.22, 36.00 | < 0.0001 |
| Olodaterol 10 µg | 121 | 389.38 (4.73) | 26.68 (4.06) | 18.69, 34.66 | < 0.0001 |
| Olodaterol 20 µg | 120 | 395.42 (4.74) | 32.72 (4.08) | 24.70, 40.73 | < 0.0001 |
| Formoterol | 123 | 387.84 (4.71) | 25.14 (4.06) | 17.17, 33.10 | < 0.0001 |
| **Evening PEF, L/min** |  |  |  |  |  |
| Placebo | 120 | 380.62 (4.64) |  |  |  |
| Olodaterol 2 µg | 119 | 391.92 (4.65) | 11.30 (4.14) | 3.16, 19.43 | 0.0066 |
| Olodaterol 5 µg | 126 | 404.70 (4.59) | 24.08 (4.07) | 16.08, 32.08 | < 0.0001 |
| Olodaterol 10 µg | 121 | 402.69 (4.63) | 22.07 (4.12) | 13.98, 30.17 | < 0.0001 |
| Olodaterol 20 µg | 120 | 406.37 (4.64) | 25.75 (4.14) | 17.62, 33.88 | < 0.0001 |
| Formoterol | 123 | 401.45 (4.61) | 20.83 (4.12) | 12.75, 28.91 | < 0.0001 |

PEF: peak expiratory flow; SE: standard error; CI: confidence interval.

**Table S7 Adverse events reported in Study 1222.6 occurring in more than one patient in any treatment group**

| **Adverse event, n (%)** | **Placebo (n = 54)** | **Olodaterol 2 µg (n = 61)** | **Olodaterol 5 µg (n = 60)** | **Olodaterol 10 µg (n = 60)** | **Olodaterol 20 µg (n = 61)** |
| --- | --- | --- | --- | --- | --- |
| Patients with adverse events | 15 (27.8) | 23 (37.7) | 24 (40.0) | 18 (30.0) | 24 (39.3) |
| Headache | 3 (5.6) | 4 (6.6) | 4 (6.7) | 3 (5.0) | 5 (8.2) |
| Nasopharyngitis | 4 (7.4) | 4 (6.6) | 5 (8.3) | 3 (5.0) | 2 (3.3) |
| Asthma | 4 (7.4) | 2 (3.3) | 2 (3.3) | 1 (1.7) | 2 (3.3) |
| Dizziness | 1 (1.9) | 3 (4.9) | 1 (1.7) | 0 (0.0) | 2 (3.3) |
| Sinusitis | 2 (3.7) | 2 (3.3) | 1 (1.7) | 2 (3.3) | 0 (0.0) |
| Pharyngolaryngeal pain | 2 (3.7) | 3 (4.9) | 1 (1.7) | 0 (0.0) | 0 (0.0) |
| Cough | 1 (1.9) | 1 (1.6) | 2 (3.3) | 1 (1.7) | 1 (1.6) |
| Tremor | 0 (0.0) | 0 (0.0) | 0 (0.0) | 0 (0.0) | 5 (8.2) |
| Respiratory tract infection | 1 (1.9) | 0 (0.0) | 1 (1.7) | 0 (0.0) | 2 (3.3) |
| Back pain | 0 (0.0) | 0 (0.0) | 2 (3.3) | 0 (0.0) | 1 (1.6) |
| Neck pain | 0 (0.0) | 1 (1.6) | 2 (3.3) | 0 (0.0) | 0 (0.0) |
| Palpitations | 0 (0.0) | 0 (0.0) | 1 (1.7) | 0 (0.0) | 2 (3.3) |
| Anxiety | 0 (0.0) | 0 (0.0) | 0 (0.0) | 0 (0.0) | 2 (3.3) |

**Table S8 Adverse events reported in Study 1222.27 with an incidence of >1% on any treatment on any system class or individual adverse event**

| **Adverse event, n (%)** | **Placebo (n = 125)** | **Olodaterol 2 µg (n = 121)** | **Olodaterol 5 µg (n = 130)** | **Olodaterol 10 µg (n = 127)** | **Olodaterol 20 µg (n = 124)** | **Formoterol (n = 125)** |
| --- | --- | --- | --- | --- | --- | --- |
| Patients with adverse events | 6 (4.8) | 12 (9.9) | 18 (13.8) | 20 (15.7) | 16 (12.9) | 8 (6.4) |
| Nasopharyngitis | 0 (0.0) | 4 (3.3) | 2 (1.5) | 3 (2.4) | 4 (3.2) | 3 (2.4) |
| Dyspnoea | 0 (0.0) | 0 (0.0) | 0 (0.0) | 2 (1.6) | 0 (0.0) | 0 (0.0) |
| Headache | 1 (0.8) | 1 (0.8) | 2 (1.5) | 3 (2.4) | 1 (0.8) | 0 (0.0) |
